# Visuospatial working memory of abacus trained and untrained children

**DOI:** 10.1371/journal.pone.0325525

**Published:** 2025-06-11

**Authors:** Evangeline Efe Ansah, Nana Yaa Nyarko, David Kwame Kumador, Justice Owusu-Bempah, Sheriffa Mahama

**Affiliations:** University of Ghana, Legon, Ghana; University Ceuma, BRAZIL

## Abstract

**Background:**

This research investigated whether there was a significant change in the visuospatial working memory of children who underwent abacus training.

**Method:**

A quasi-experimental research design with a quantitative approach was employed for the study. Ninety (90) children were recruited as the experimental group and one hundred (100) children as the control group. The experimental group participated in abacus training as part of their extracurricular activities at school. A questionnaire assessing visuospatial memory was administered to both groups before and after the training.

**Findings:**

The findings revealed a significant change in the mean scores of the experimental group from before and after the abacus training (p < .01) compared to the control group. This signified that abacus training may potentially lead to significant improvement in visuospatial working memory.

**Conclusion:**

It is suggested that future research be conducted using a longitudinal approach to enable a more comprehensive assessment of the full course of abacus training.

## Introduction

The abacus is a tool used to perform basic arithmetic operations in various number systems [1]. It consists of vertical rods on which beads move up and down and a beam that divides the upper and lower portions. The beads on the abacus represent numbers in a visuospatial format, which facilitates mental arithmetic [2]. Abacus users who have been trained for some time acquire abacus-based mental calculation skills and can perform fast and accurate calculations by manipulating an imaginary abacus in their minds. Research has found that the aspect of memory that facilitates this fast and accurate mental calculation is the visuospatial working memory [3–5], an aspect of cognition and knowledge that enables this mental ability over time and practice [6–8].

### Visuospatial working memory (VSWM)

Visuospatial working memory is the ability to hold and process an object’s spatial information and location in the mind, an essential component of working memory and used for many daily tasks [9]. As a component of working memory, VSWM is in control of the recall of stimuli with visual and spatial interaction, as well as retaining and manipulating such information [10]. Assumed to be responsible for processing and the short-term storage of visual and spatial information [11,12], VSWM divides its functions into active and passive functions [13]. The passive functions refer to visuospatial information being stored for a short term and are assumed to comprise two separate storage components: storage of simultaneous visuospatial information, such as colour, shape and size, while the other storage is for visuospatial sequential information, such as movement sequences. On the other hand, the active functions denote the processing functions that can also be characterised as central executive functions [14–18].

VSWM, as a multiple-component cognitive system, comprises a central executive and two sub-systems: the phonological loop and the visuospatial sketchpad. The phonological loop is known to be involved in the processing and holding of verbal information, while the visuospatial sketchpad is needed in processing and holding visuospatial information [19]. The acquisition of VSWM skills facilitates letter/number recognition, reading, writing and math for young children and involves the ability to remember shapes and colours and their locations and movements [20,21]. This skill of VSWM helps to facilitate abacus mental arithmetic, with speed and accuracy.

### Abacus training

The Abacus is a traditional device used to facilitate arithmetic operations and represents numbers by the visuospatial locations of beads. It is commonly used in Asian countries to facilitate arithmetic operations, namely addition, subtraction, multiplication, division and root calculations and has gone through quite a few changes from its earliest form as a sand table with pebbles as counters into its current design with beads that move on rods [22]. The most widely used abacus in the world is the soroban [23], which has two main parts: the upper part, also known as “heaven beads”, and the lower part, also called “earth beads”. The heaven beads have a value of five, while the earth beads have a value of one bead each [24]. The representation of numbers in a visuospatial format makes it easier to use for basic operations as well as the extraction of square and cube roots [25].

Some individuals can train and use the abacus to perform arithmetic calculations mentally in a fast but accurate manner. This technique of mentally using the abacus is known as Abacus-based Mental Calculations (AMC). Abacus learners skilled in performing mental calculations with speed and accuracy not only employ the use of a physical abacus but also manipulate an imaginary abacus in their mind [26]. This AMC skill is acquired through intensive training over a period, during which learners over time and with training move from operating beads on a physical abacus to manipulating only imaginary abacus beads in their minds with speed and accuracy by moving fingers in the air. AMC is an advanced technique learned by abacus users that enables them to visualise the physical abacus and perform fast and accurate mental calculations after consistently practising for a period [23].

### Relationship between VSWM and abacus training

Earlier research has indicated that math abilities play a significant role in one’s academic success [27], career aspirations [28,29], judgment and decision-making [30], market and non-market outcomes [30,31]. These skills are a factor of executive function, composed of working memory and task switching [32,33]. Working memory refers to the maintenance and manipulation of goal-directed information over short periods [34]. Working memory is assumed to be formed by a central executive control system that monitors two independent subsystems: the visuospatial sketchpad or visuospatial working memory for spatial processing and the phonological loop for non-spatial, mostly linguistic information processing [35].

Studies on visuospatial working memory have proposed that the usage of the storage and manipulation processes of visuospatial working memory is a visuospatial strategy, which is the basis of Abacus-based mental calculation [3–5]. Further studies have revealed that although the orthodox arithmetic operation employs brain regions related to the phonological working memory and other language-related areas [36], AMC engages brain networks that largely overlap with the primary neural substrate of VSWM [4,5,37].

Mental skills training enhances brain plasticity, particularly in functional brain activation, connectivity, and anatomy [38,39]. Several studies indicate that Arithmetic Mental Calculation (AMC) promotes arithmetic literacy and improves cognitive abilities [40,41]. In this context, Wang et al. in 2015 [42] found that 7-year-old children who learned AMC performed better in math compared to their peers in the control group, particularly in arithmetic and visual-spatial domains. Further, Wang et al. in 2019 [8] demonstrated that long-term AMC not only enhances arithmetic ability but also improves Visuospatial Working Memory (VSWM) performance in children. They attributed this improvement to functional changes in the frontal, parietal, and occipital areas of the brain, highlighting the significant role of the middle frontal region in transferring gains from AMC training to VSWM. In 2016, Dong et al. [43] concluded that the impact of AMC training on VSWM might be due to more efficient execution of VSWM-related cognitive processes facilitated by optimising neural resources, especially in visuospatial areas. During calculation, the visuospatial sketchpad acts as a mental writing board for encoding, retaining, and manipulating information [44]. Supporting this concept, researchers have found that the visuospatial sketchpad significantly correlates with and predicts children’s arithmetic development [45,46]. Additionally, Syväoja et al. in 2021 [47] showed a moderate association between VSWM and math competence, motor skills, and various other measures of cognitive functioning in students from grades 6–9 in their studies.

### Aim

Research indicates that abacus use for mental calculations is prevalent in Asian nations, where users often excel in mental arithmetic compared to their Western counterparts [48]. Despite evidence of the benefits of abacus training, empirical data on its impact in countries, such as Ghana, remains scarce. This paper evaluates the visuospatial working memory of children trained in abacus versus those who are untrained, hypothesising a significant improvement in the former group.

## Methods

### Respondents and procedure

Respondents were purposively sampled from four schools in New Achimota, a suburb of Achimota township in the Greater Accra region of Ghana. The study employed a quasi-experimental design conducted over four months, from June to September 2021, with a sample of one hundred children from primary 4 to primary 6. The sample size for the study was determined by the use of the Yamane [49] formula for a known population:


$$n\;=\;\frac{N}{1+N{\;(e)}^{2}}$$

where ‘n’ and ‘N’ are the sample and population sizes, respectively. The letter ‘e’ is the level of precision (Isaac, 2003).

In the present study, the total number of school children considered, ‘N’, was 420, and ‘e’ was estimated at 0.05.

Therefore, the sample size, $n\;=\;\frac{420}{1+420{\;(0.05)}^{2}}=204.88\approx 205$.

Following that, the researcher selected a sample size of 200 children for the investigation, including an experimental group of fifty children each receiving abacus training at two schools and a control group of fifty children each from two schools without abacus training. Ten children from the experimental group dropped out during the study. Written consent was obtained from schools, parents and participants. Both control and experimental groups received the Ghana Education Service’s ‘Standard-Based Curriculum (SBC)’, which is the standard for all basic schools in Ghana. The SBC, started in 2019, emphasises reading, writing, arithmetic, and creativity as predicates for the development of 21st-century skills, including critical thinking and problem-solving [50]. The children, after 6 years of basic school and 3 more years of junior high school, take the national Basic Education Certificate Examination (BECE).

The experimental group, in addition to the regular school curriculum over the course of the 4 months, received an additional two hours of weekly abacus training. No prior cognitive tests and assessments for disabilities or learning difficulties were carried out since the groups received the same SBC curriculum and were considered equivalent in all aspects, with the only difference being the abacus training. Only the tests mentioned below were used in subsequent analysis and results; no classwork or other performance measures were considered. The tests used were administered in the participants’ classrooms within thirty minutes at the beginning and end of the four-month training. The Ethical Committee for Basic and Applied Sciences at the University of Ghana, Legon, reviewed and approved the ethical considerations of the study (research protocol code ECBAS 032/20–21). Respondent ages ranged from 9 to 11 years, with a mean age of approximately 10.

### Inclusion and exclusion criteria for experimental and control groups

For the experimental group, the inclusion criteria were: (1) children in the target age in classes, primary 4, 5, and 6, children who took part in the abacus training regularly for a minimum of 4 months, because it takes three to four months to complete the basics as confirmed by Vasuki [51], (2) children whose parents gave and signed the consent to satisfy ethical requirements and (3) children who assented to take part in the study. The exclusion criteria were: (1) children who dropped out of the abacus program during the training and (2) children who joined the abacus program in the middle of the study period, because the student may not have fully grasped all the basic level skills.

For the control group, children who met all the three inclusion criteria were selected: (1) children in the target age in primary (levels 4, 5, and 6) who did not take part in the abacus training, and (2) children whose parents gave and signed the consent to satisfy ethical requirements, (3) children who assented to take part in the study. The exclusion criteria were (1) children who joined the class in the middle of the term so did not fulfil the four-month requirement. This is also important for equivalence between the two groups and (2) children who dropped out of school during the study period. The background characteristics of the respondents are shown in Table 1.

**Table 1:** Demographic characteristics of respondents.

Characteristics	Frequency (n)	Percentage (%)
**Gender**		
Male	85	44.50
Female	105	55.50
**Age (years)**		
9	40	21.1
10	60	31.6
11	90	47.4
**Schools**		
School A	80	42.1
School B	20	10.5
School C	56	29.5
School D	34	17.9
**Class**		
Class 4	57	30.5
Class 5	68	35.8
Class 6	65	33.7
**Groups**		
Experimental group	90	47.4
Control group	100	52.6

### Measures

#### Raven’s colored standard progressive matrices (RCPM).

Raven’s Colored Progressive Matrices is a standardised test for visuospatial perception, which requires participants to match a target item to a corresponding pattern [52]. The test consists of three sub-tests (A, AB, and B), each containing twelve (12) items. Respondents are presented with coloured matrices, each featuring one missing part, from which they must select the correct option out of six. The sub-tests, totaling thirty-six (36) items, are designed in increasing order of difficulty, enabling respondents to employ observation and analytical reasoning strategies to complete the missing matrices. Specifically, Set A assesses pattern recognition, Set AB evaluates spatial perception, and Set B measures abstract thinking. Previous studies utilising Raven’s Matrices in Ghana reported a Cronbach’s alpha of 0.79 [53], while the current study yielded an alpha of 0.86.

#### Digit memory test.

The digit memory assessment is evaluated for both forward and backward recall of digit sequences, with the forward recall allowing for a maximum of nine digits and the backward recall permitting a maximum of eight digits [54]. According to Ryan and Lopez [55], forward-digit recall assesses auditory attention and sequencing, while backward-digit recall examines mental tracking, short-term memory, and visuospatial imagery. Participants are required to recall a series of numbers presented to them, and the aggregate count of correctly recalled digit sequences from both forward and backward spans is used to determine the total correct score. The Cronbach’s alpha for the current sample was reported to be 0.74

#### Letter cancellation test (LCT).

The letter cancellation test is a paper and pencil test with rows of letters randomly interspersed with a designated target letter and requires learners to scan, locate and cross out the target letters. This test assesses selective attention, concentration and visuospatial scanning abilities [56,57]. Performance was scored according to the number of correct target letters crossed out [58]. The Cronbach’s alpha obtained for the current sample was 0.53.

### Analytical procedure

The data collected in both pre-test and post-test questionnaires were analysed using SPSS version 23 software. The independent t-test technique was used to test for the significance of mean differences in the experimental and control groups.

## Results

### Respondents’ overall performance on the three tests

The sum of respondents’ scores for each of the three tests, for both pre and post-test, was grouped into poor, low, average and high ranges, as shown in Table 2.

**Table 2:** Overall performance on the three tests.

Category (range)	Pretest	%	Posttest	%
**Poor (1–19)**	0	0	0	0
**Low (20–38)**	3	1.6	1	0.5
**Average (39–57)**	85	45.7	69	36.3
**High (57–76)**	102	52.7	120	63.2
**Total**	190	100	190	100

### Respondents’ performance on Raven’s Colored Progressive Matrices Test (RCPM)

The total scores of the RCPM test 36 were divided by three (3) intervals and used to create ranges, namely, low, average and high ranges (Table 3). For the experimental group, there was a 10% decrease in posttest results for those who had average scores. There was a 7.8% increase in those who had high scores. The control group, on the other hand, showed a 6% decrease in the average score and an 8% increase in those with high scores.

**Table 3:** Performance on the Raven’s Colored Progressive Matrices test (RCPM).

Category (range)	Experimental Group		Control Group	
	Pretest (%)	Posttest (%)	Pretest (%)	Posttest (%)
**Low (1–12)**	0	2 (2.2)	2 (2)	0
**Average (13–24)**	40 (44.4)	31 (34.4)	46 (46)	40 (40)
**High (25–36)**	50 (55.6)	57 (63.4)	52 (52)	60 (60)
**Total**	90 (100)	90 (100)	100 (100)	100(100)

### Respondents’ performance on the Digit Memory Test (DMT)

The total scores of DMT 30 were divided by three (3) intervals and used to create ranges, which were designated as low, average and high in Table 4. There was an improvement in the low score category of the experimental group with a 10% decrease at posttest as compared to the control group, which remained unchanged. At posttest, the experimental group saw an increase of 7.7% at the average score level and 2.3% at the high score level, which means that progress was made at the end of the four months of research. Inversely, the control group saw no changes at both the average and high scores levels since the number hence percentages remained the same.

**Table 4:** Performance on the Digit Memory Test (DMT).

Category (range)	Experimental Group		Control Group	
	Pretest (%)	Posttest (%)	Pretest (%)	Posttest (%)
**Low (1–10)**	10 (11.1)	1 (1.1)	10 (10)	10 (10)
**Average (11–20)**	77(85.6)	84 (93.3)	87 (87)	87 (87)
**High (21–30)**	3 (3.3)	5 (5.6)	3 (3)	3 (3)
**Total**	90 (100)	90 (100)	100 (100)	100 (100)

### Respondents’ performance on the Letter Cancellation Test (LCT)

The results showed that the performance of the experimental and control groups on the Letter Cancellation Test (LCT) did not differ in any meaningful way between the pretest and posttest across the three categories, as presented in Table 5.

**Table 5:** Performance on the Letter Cancellation Test (LCT).

Category (range)	Experimental Group		Control Group	
	Pretest (%)	Posttest (%)	Pretest (%)	Posttest (%)
Low (0–6)	0	0	1 (1)	0
Average (7–13)	1 (1.1)	1 (1.1)	2 (2)	1 (1)
High (14–20)	89 (98.9)	89 (98.9)	97 (97)	98 (98)
Total	90 (100)	90 (100)	100 (100)	100 (100)

### Difference between the visuospatial working memory of abacus-trained and untrained schoolchildren

To determine whether there was a statistically significant difference in the means of the experimental and control groups after four months, an independent sample t-test was conducted (Table 6). The results showed a significant difference in the experimental group with a *p-value* of 0.000. However, they were not significant for the control group (*p* = 0.09). Therefore, the hypothesis, suggesting a significant change in visuospatial working memory after an abacus training, was confirmed, albeit with small effect sizes [59,60]. The difference in means for visuospatial working memory, as measured by RCPM (VSWMRCPM), was 1.63 (*p* = 0.45) for the experimental group and 1.27 (*p* = 0.45) for the control group. Meanwhile, the mean difference for visuospatial working memory as measured by DMT (VSWMDMT) was 0.88 (*p* = 0.001) for the experimental group and 0.01 (*p* = 0.58) for the control group.

**Table 6:** Differences within experimental and control groups.

	Experimental							Control							Effect Size *d*_*ppc2*_
	Pre		Post					Pre		Post					
	M	SD	M	SD	Diff	*t*	Sig	M	SD	M	SD	Diff	*t*	Sig	
**VSWM**_**RCPM**_	24.68	5.06	26.68	5.75	1.63	−2.02	***.45***	24.93	5.75	26.20	6.28	1.27	−1.37	*0.17*	0.13
**VSWM**_**DMT**_	14.36	3.29	16.20	3.16	1.84	−3.42	***.001***	14.32	3.16	14.33	3.17	0.01	0.56	*0.58*	0.57
**VSWM**_**LCT**_	18.93	1.67	19.52	1.23	0.59	−2.23	***.03***	18.65	2.25	19.20	1.49	0.55	−1.92	*0.56*	0.03
**VSWM**_**TOTAL**_	57.97	6.39	62.03	7.78	4.06	−3.93	***.000***	57.88	7.88	59.73	7.88	1.85	−1.66	***0.09***	0.31

## Discussion

This paper presents a thorough investigation of the effects of abacus training on school-aged children’s visuospatial working memory (VSWM), with a specific focus on performance between those who had four-month abacus training and those who did not. The findings demonstrated statistically significant improvements in performance on Raven’s Colored Progressive Matrices (RCPM) across the experimental and control groups, suggesting that participants from both cohorts experienced enhanced capacities for abstract reasoning. This enhancement was particularly evident in their ability to recognise relationships within matrix puzzles and to identify missing components during post-test evaluations.

These results resonate with prior research, including the work conducted by Tandoh et al. [61], which recorded similar performance metrics among school-aged children (ages 9–12) from disparate communities in eastern Ghana. Notably, significant divergences were observed in the Digit Memory Test (DMT), where the experimental group exhibited pronounced enhancements in the recall of digits in both forward and backward sequences, clearly surpassing the performance of the control group. Such improvements can be attributed to the distinctive cognitive strategies employed by learners engaged in abacus training, as articulated by Bhaskaran et al. [62]. This research posits that learners can conceptualise numbers on an imaginary abacus, allowing them to manipulate numerical relationships mentally. This indicates the development of superior recall abilities compared to peers who did not participate in abacus training.

Moreover, it is crucial to acknowledge that both groups demonstrated commendable performance on the letter cancellation test, thereby indicating that the benefits of cognitive training transcend specific memory tasks. Overall, this study highlights the potential advantages of integrating abacus training into educational curricula to enrich cognitive skills and support academic achievement among young learners. Such findings provide a compelling argument for exploring and integrating innovative pedagogical methodologies that could significantly influence cognitive development in children.

Statistical analysis utilising independent sample *t*-tests showed general improvements across both groups following the 4-month training period. However, comparisons indicated a significant mean change in the experimental group alone. The difference in means for visuospatial working memory, as measured by RCPM, yielded a non-significant result for both groups. This finding implies that the experimental group demonstrated a marginal improvement in their ability to perceive spatial arrangements and engage in abstract problem-solving within matrix contexts compared to the control group. Conversely, the difference in means regarding visuospatial working memory, as assessed by DMT, was highly significant in favour of the experimental group compared to the control group. The components of the Digit Memory Test—auditory sequencing, mental tracking, and visuospatial imaging—demonstrated a marked increase in these cognitive skills within the experimental group relative to their peers.

Supporting these findings, Dong et al. [43] noted significant memory spans from pretest to posttest in the abacus group, attributing improvements to the necessity during mental arithmetic exercises to retain an increasing quantity of beads while simultaneously utilising both visuospatial and phonetic representations of numbers. This dual cognitive engagement facilitates enhanced listening and recall capabilities in the digit memory assessment.

When comparing the difference in means of visuospatial working memory as evaluated by the Letter Cancellation Test (VSWMLCT), it was evident that the experimental group exhibited significantly greater performance than the control group. The consistent practice of manipulating physical beads on an abacus engages the learners’ visuospatial working memory by forming mental images of the abacus alongside the positioning and movement of beads and rods. Over time, as learners master physical abacus usage, they cultivate a mental model of an imaginary abacus that can be visualised and manipulated to derive computational results.

Participants adeptly listen to numerical inputs for calculations, simulate movement on the imaginary abacus, and rely on their visuospatial working memory to articulate answers swiftly and accurately [62]. Wang [2] expounded on this notion by stating that the representation of the abacus in a visuospatial framework enables learners to execute mental arithmetic using an imagined abacus. This understanding elucidates why nearly all differences in means for the experimental group were statistically significant. At a p-value of 0.000 (p < .01), the overall mean differences, aggregating results from all three tests, were significantly different for the experimental group, underscoring that after a 4-month intervention period, abacus training substantially enhanced the visuospatial working memory of school children [38,39,43,60].

## Conclusion, implications and limitations

Drawing from the study’s findings, it can be concluded that training children to use the abacus for mental arithmetic enhances their calculation skills and significantly but marginally boosts their visuospatial working memory. Although the duration of the training was limited to a specific timeframe, the observable improvements in children’s mathematical abilities suggest that even short-term exposure to abacus training can yield some cognitive benefits. This reinforces the notion that such educational techniques possess considerable potential for enriching learning environments, particularly as they provide an engaging method to enhance cognitive functions among young learners.

In terms of implications, the findings from this study provide empirical evidence that is relevant for schools, parents and other stakeholders who are seeking effective methods for math learning and positive, longer-term learning outcomes. This study provides evidence, albeit basic and short-term, on the potential positive impact of abacus training on the VSWM of school-aged children, which can stimulate further interest and research. Furthermore, it is essential to note that the study primarily examined basic abacus training, indicating a notable gap in existing research regarding the long-term impacts and effectiveness of more advanced abacus use. Future research in this area could greatly benefit from a larger sample size and a longitudinal approach. Such an approach would involve participants undergoing comprehensive abacus training over an extended period, perhaps several months or even years. This could help ascertain the sustained cognitive benefits of abacus training, its generalizability and its effectiveness across various age groups and learning environments. Also, future studies could look at how other non-human learning-support artefacts and models, such as computing, gaming, and artificial intelligence, and culturally sensitive games such as ‘Ghanaian draught or checkers’, ‘ludo’ and ‘ampe’ could benefit the development of VSWM in school-aged children.

The methodology, although robust, does not provide information on how abacus training can be more seamlessly integrated into the regular school curriculum without compromising time for other pursuits. By building upon these initial findings, researchers are encouraged to formulate more nuanced strategies for integrating abacus training into school curricula. Such enhancements could promote the development of children’s cognitive skills more effectively. Comprehensive studies exploring diverse methodologies and frameworks for abacus instruction might lead to a robust understanding of its role in fostering a more profound cognitive skill set among children, thereby shaping future educational practices.

### Statement of significance

**Table:** 

Problem or Issue	Does abacus training comparatively lead to significant improvement in visuospatial working memory.
What is Already Known	Schools and clubs around the world but mostly in Asia and increasingly in Ghana ascribe to abacus training to help in math learning and general academic development.
What this Paper Adds	Empirical research using a quasi-experimental design to assess whether abacus training leads to significant visuospatial working memory improvement

## Supporting information

S1 File Abacus pre.posttest data Ghana 2021. (SAV)
